# Clinical Outcome of Fully Digital Workflow for Single-Implant-Supported Crowns: A Retrospective Clinical Study

**DOI:** 10.3390/dj10080139

**Published:** 2022-07-27

**Authors:** Francesco Gianfreda, Paolo Pesce, Erich Marcano, Valeria Pistilli, Patrizio Bollero, Luigi Canullo

**Affiliations:** 1 Department of Industrial Engineering, University of Rome “Tor Vergata”, 00133 Rome, Italy; francesco.gianfreda@ptvonline.it; 2 Department of Surgical Sciences (DISC), University of Genoa, Ospedale S. Martino, L. Rosanna Benzi 10, 16132 Genoa, Italy; paolo.pesce@unige.it; 3 Co-Tutela Modality, Central University of Venezuela, Caracas 1040, Venezuela; erichmarcano@gmail.com; 4 Independent Researcher, 00198 Rome, Italy; valeria.pistilli93@gmail.com; 5 Department of System Medicine, University of Rome “Tor Vergata”, 00133 Rome, Italy; patrizio.bollero@ptvonline.it

**Keywords:** fixed-prosthodontics, digital impression, intraoral scanner, full digital workflow, guided implant surgery, CAD-CAM, zirconia

## Abstract

A digital workflow by means of intraoral scanners and computer tomography has been used in dental implantology, allowing clinicians to be potentially more accurate and precise. Computer-Aided Design and Computer-Aided Manufacturing (CAD-CAM) and 3D models facilitate the process from treatment planning to the surgical procedure, up to the implant placement and final prosthesis. The aim of the present retrospective study was to evaluate a fully digital workflow for single-tooth implant rehabilitation. A total of 19 patients (22 implants) were included in the present study, with a mean follow-up time of 2 years. A fully digital workflow was performed on each patient through the planning, design and printing of a surgical guide, following a digital impression made with an intraoral scanner, computer-tomography-guided implant placement and, finally, with the delivery of a CAD-CAM crown. The two-year follow-up results were satisfactory in terms of the aesthetic yield and precision of the prosthesis. In single-implant-supported restorations, due to digital protocols and digital planning, a reduced number of clinical sessions was registered and the treatment plan results were more predictable. Future studies are needed to understand the application of fully digital protocols in cases of partially or totally edentulous patients.

## 1. Introduction

Innovative materials and technologies used to improve treatment outcomes, reducing, at the same, time morbidity, biological and surgical times, are an intense research topic in dentistry [1]. The full-digital workflow in single-tooth implant rehabilitation is now a reality, allowing dentists to plan digital treatments and obtain simpler and faster clinical procedures with predictable results. Nevertheless, there are still some inaccuracies that can result in treatment failure. The learning curve is an important factor in the predictable results [2]. The use of a digital workflow with computer-aided manufacturing (CAM) gives dental clinicians several advantages [3]. Within these, a complete digital workflow is preferred by patients and operators [4]. Using digital technology, clinicians can plan from surgery to final prosthesis using 3D models and CAD-CAM machines [5]. The complete digital workflow includes digital impressions, 3D radiographic scanning, digital planning and the digital design and milling of the prostheses [4]. The digital workflow in dental implantology has been used for treatment planning by means of intraoral scanners and computer tomography, allowing clinicians to be potentially more accurate and precise when using guided implant surgery [6,7]. Digital impressions seem to have several advantages in implant rehabilitations, such as the re-scanning of a defected area, immediate sharing with the dental technician and more comfort for patients, with better patient acceptance [3,8]. Although some dental operators are not motivated to change from a well-known and moderate-cost technique to an unknown and expensive one, current impressions and materials are now excellent and simple to use [9,10]. The digital 3D models give dentists precise and complete anatomical information, including the soft tissues contour [10,11]. From the 3D model, a stereolithographic surgical guide for 3D static guided placement can be designed and printed [2]. The use of guided implant placement, based on computer tomography and digital planning, should save time and decrease risks during surgery [12]. 

Milled crowns for single implants seem to allow precise and accurate restorations [13]. Either dental lab or chairside milling machines offer reasonable time saving, but with different levels of esthetic characteristics [14]. However, the high investment, long learning curve and inconsistent high-level scientific literature often represent major obstacles [4] to the transition from an analogical to fully digital workflow for dentists. The aim of the present study is to evaluate a fully digital workflow for single-tooth implant rehabilitations in order to add evidence in this specific field of restorative dentistry.

## 2. Materials and Methods

### 2.1. Study Design

The present study is a retrospective case series study conducted in one clinical center in accordance with the Good Clinical Practice Guidelines (GCPs) following the recommendations of the World Medical Association Declaration of Helsinki ethical principles for medical research involving human subjects as revised in Fortaleza (2013). The retrospective study was registered on ClinicalTrials.gov (Identifier: NCT05011604). The study was approved by the ethical committee of the University of Genova (Statement N. 2021/62). All patients were informed about the benefits and the possible risks of a fully digital workflow and its alternatives. A signed written consent was obtained for each patient.

### 2.2. Subject Population

The current retrospective study was performed in a private clinic in Rome, Italy.

Inclusion criteria were:

1. Patients over 18 years of age;
2. Good general health conditions according to the American Society of Anesthesiology scale (ASA 1 or ASA 2) [15];
3. Patients requiring single-implant-supported reconstructions.

Patients were consecutively enrolled in the present study and were treated by the same operator (LC) between January 2017 and June 2020.

Exclusion criteria were:

1. General contraindications to oral surgery;
2. Periodontally compromised patients (probing pocket depth ≥ 5 mm, bleeding on probing, and plaque index ≥ 25%);
3. Patients requiring bone augmentation procedures.

### 2.3. Surgical and Prosthetic Workflow

**Figure 1:**
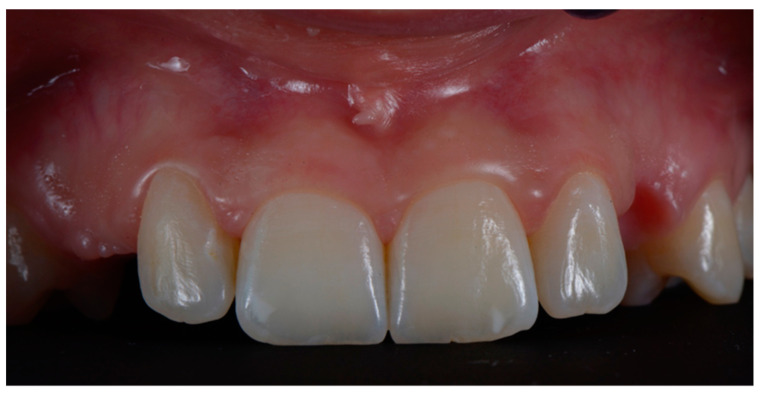
Case of edentulous upper canines following surgical extraction of the impacted elements. Preliminary photo before surgical treatment.

**Figure 2:**
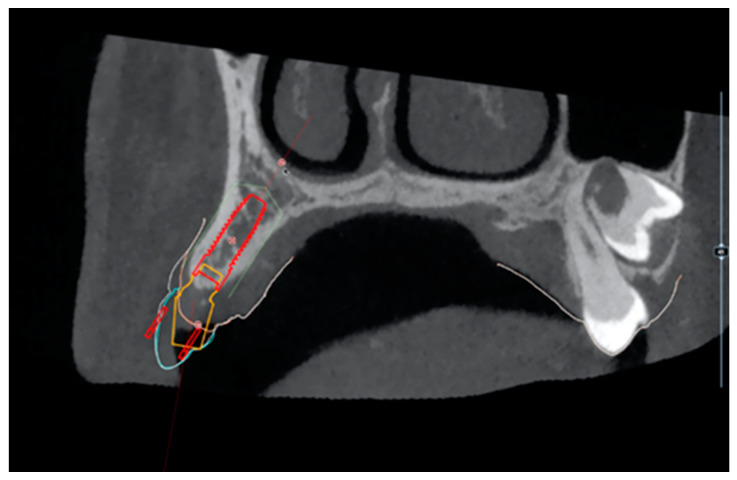
Guided implant planning in zone 1.3 using CBCT, the soft tissue contour, and the digital wax-up. Computer-guided planning of abutment angulation was made according to the digital diagnostic wax-up of the 1.3 element.

For all patients included in the present study, a digital impression was made using an intraoral scanner (CS3600, Carestream Dental, Atlanta, GA, USA) following standard protocols described in literature [16]. The same day, a CBCT (CS9000 3D, Carestream Dental, Atlanta, GA, USA) scan was performed on the mandibular or maxillary jaw with the missing tooth. The STL and the DICOM files were then imported and superimposed to allow for the surgical planning (RealGuide, 3Diemme, Cantù, Italy) (Figure 1 and Figure 2).

**Figure 3:**
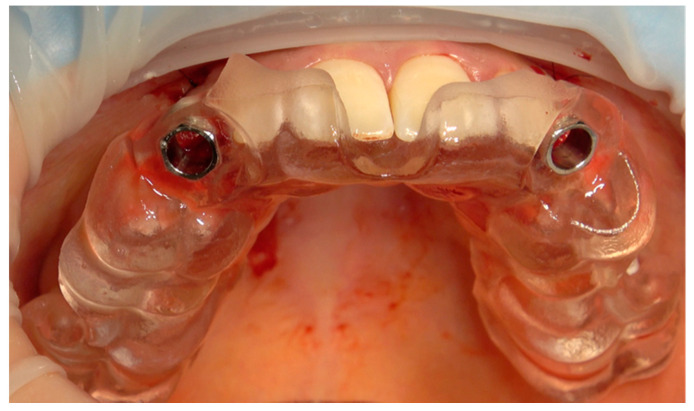
Surgical guide template.

**Figure 4:**
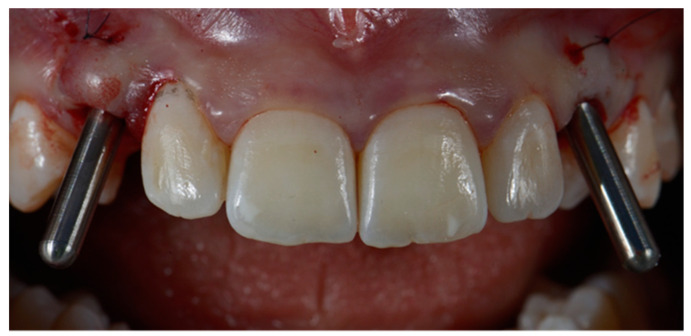
Implants insertion with positioning pins.

One week after planning, the flap was elevated with a papilla preservation technique, and guided implant placement was performed. Once designed, the flap was disepitelized using a diamond bur. Then, the flap was elevated to expose bone. Soft tissues were reflected with a roll-flap with the aim of increasing the volume on the buccal side. Implant (Sweden & Martina, Padua, Italy) was then inserted, and a digital impression with implant-level PEEK scan bodies was made. The healing abutments were screwed and a 6.0 suture was positioned (monofilament-polyglecaprone suture, Surgiclryl-Monofast, ^®^SMI-Belgium) to stabilize the soft tissues (Figure 3 and Figure 4). 

**Figure 5:**
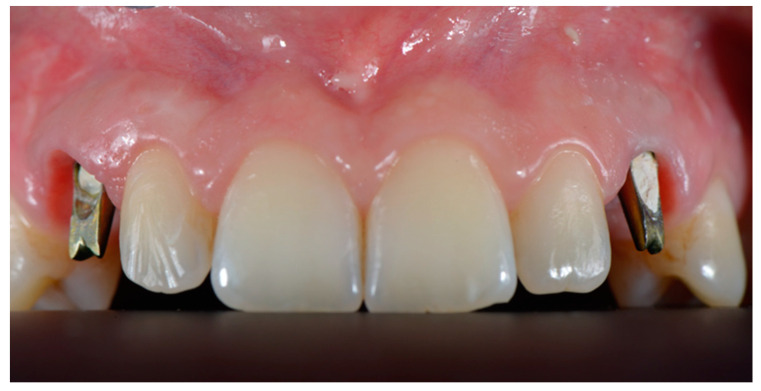
Definitive CAD-CAM milled abutments.

The file of the impression was sent to the dental technician and a final custom milled abutment (Sweden & Martina, Padua, Italy) and a provisional restoration were designed and manufactured using CAD-CAM technologies [17]. One week later, definitive abutment was positioned and screwed at 25 N/cm (Figure 5). 

**Figure 6:**
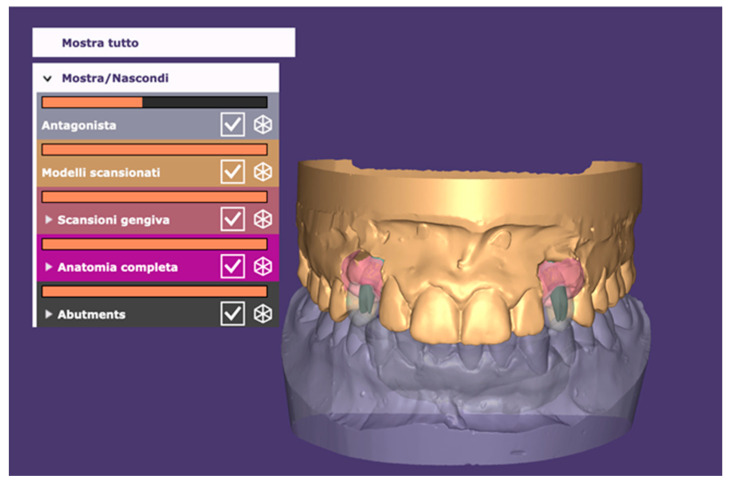
CAD-CAM restorations.

Provisional restoration was then cemented using temporary cement (Temp Bond, Kerr Sybron Dental Specialities, Washington, DC, USA), removing all of the possible occlusal contacts both in protrusive and lateral positions. Sutures were removed 2 weeks after placement. Three months thereafter, once osseointegration was obtained, intra-oral scans were performed focused on the temporary inserts with and without the crowns. Finally, the emergence profile of the temporaries was also scanned to avoid the collapse of the tissues around the abutments without crown. In this way, the laboratory was able to match all of the scans made together with the CAD-CAM project of the abutments already inserted to produce the final crowns in monolithic zirconia (Figure 6). 

Eventual deficiencies of the prosthetic rehabilitation at the time of provisional restoration, such as contact points and esthetics, were noticed and fixed by directly adding composite. In all cases, a new impression after soft tissue maturation was performed and strategically, a new crown in polymethyl methacrylate (PMMA) was designed and realized. 

The crown with the final PMMA shape was used to evaluate the final structure and emergence profile of the crown. Any PMMA modifications required were conducted chairside. The subsequent PMMA scan determined the final shape of the zirconia prosthesis delivered by the technician.

**Figure 7:**
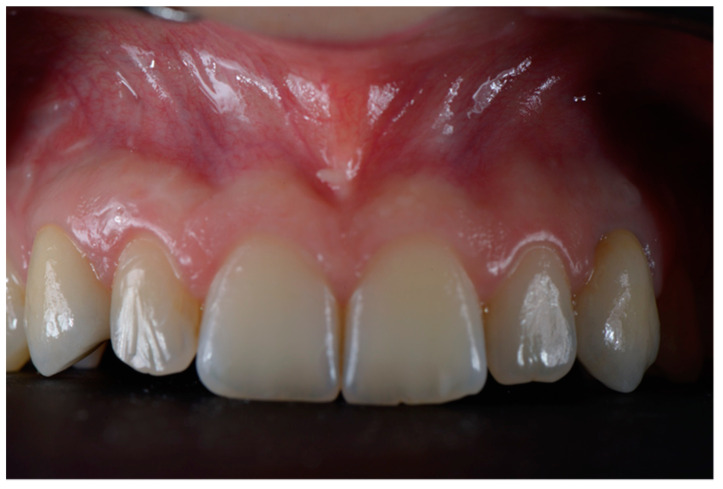
Definitive restorations after 2-year follow-up.

**Figure 8:**
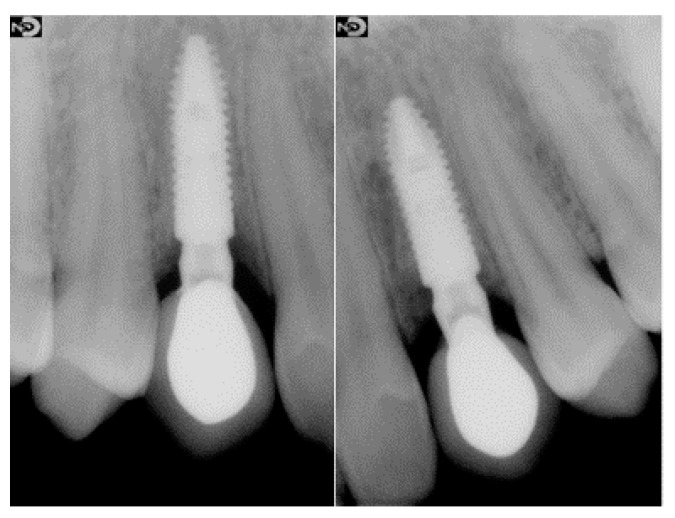
Radiographs of definitive restorations after 2-year follow-up.

Final check before producing the zirconia element was performed positioning the crown on the abutment. If no color or shape corrections were needed, definitive zirconia restoration was positioned and cemented (Temp Bond, Kerr, FL, USA) (Figure 7 and Figure 8).

### 2.4. Outcome

Data collection and processing were carried out with Excel 2019 (Microsoft Corporation, Washington, DC, USA). The descriptive analysis took into consideration the following outcomes: demographic characteristics, positions of the implants to be inserted, number of appointments necessary for the definitive delivery of the prosthesis, and number of adjustments necessary before delivery of the prosthesis. The follow-up of the various cases was also analyzed. Bleeding on Probing (BP), Plaque Index (PI), and Probing Depth (PD) were evaluated as bleeding using a probe PCPUNC15 (Hu-Friedy, Chicago, IL, USA) around the peri-implant mucosa. 

Marginal Bone Loss (MBL) was evaluated using intraoral radiographs taken at each follow-up visit. All radiographs were digitally analyzed using an image-processing software program (Adobe Illustrator 2021; Adobe Systems, San Jose, CA, USA).

In each patient, it was evaluated whether it was possible to carry out immediate loading considering an insertion torque greater than 35 Ncm as a positive value.

In addition, to evaluate the soft tissue contours, the Pink Esthetic Score (PES) was calculated as described by Furhauser et al. [17] in the intercanine zone.

## 3. Results

A total of 19 patients (22 implants) were included in the present study, with a mean follow-up time of 2 years (25.3 months, range 24–30). The main demographic information is reported in Table 1.

Fifteen patients required 18 implants inserted in the upper arch, and four patients required 4 implants in the lower arch.

All cases were performed on healed sites. In all cases, an insertion torque higher than 35 Ncm was obtained, which allowed for the insertion of a temporary abutment after one week.

All implants were rehabilitated with edgeless milled CAD-CAM abutments. The temporary crowns were all made in CAD-CAM using PMMA resin discs. In all situations, a relining procedure with the flow composite was performed to condition the soft tissues. All implants were restored with single stratified zirconia cement-retained crowns. Only in four cases did the PMMA restoration require minimal adjustments by the technician chairside. Overall, the average number of appointments required to complete the single rehabilitation with the described protocol was 6.21. All implants resulted in being osseointegrated at the end of the study follow-up. No prosthetic complications resulted during the study, except for one minimal ceramic chipping on one premolar site. The problem was solved by finishing the crown. No esthetic outcome was reported. For all patients, periodontal parameters resulted as being healthy (PD lower than 3 mm, BoP < 25%, PI < 20%). The mean MBL was 0.28 mm (SD: 0.14) and showed no significant differences related to the treated site.

The PES of the elements from canine to canine showed a heterogenicity of the values (min 2–max 14) often due to the baseline situation before the intervention. Finally, the average PES was 9.31, with a standard deviation of 3.92. 

It is important to underline that these results derive from a graftless approach in which no bone or soft tissue regenerative interventions were used, except for the roll-flap in the implant insertion phase.

## 4. Discussion

The aim of the present retrospective study was to evaluate an implant-supported fully digital workflow for the single tooth replacement by means of an implant-supported crown. The results seem to indicate a great predictability in intercalated edentulism cases. By breaking down the total workflow into the various phases, it is possible to observe how digital surgery planning can be safe and predictable. Through the matching between the DICOM and STL files, digital planning allows for the three-dimensional evaluation of the present bone and the pre-operative planning of the future prosthetic rehabilitation [16]. Various protocols have been proposed in the literature to transfer the information of the diagnostic wax-up of radiographic examinations [18]. A ‘fully digital’ prosthetic project can be carried out directly with the planning software or by matching a digital wax-up in STL format with the corresponding DICOM file. Instead, a final not ‘fully digital’ protocol can transfer the traditional diagnostic wax-up through a CBCT-exam [19]. The use of dental-supported surgical guides in cases of mono-edentulousness guarantees a greater stability and precision than all other templates with mixed or mucous support [20]. It is important to remember that each procedure contains its own margin of error, which becomes more relevant in more complex cases [21]. In this fully digital protocol, the implants were also inserted with a surgical guide by means of special inserts that allowed the surgeon to predetermine the exact depth of the fixtures [22]. 

After implant insertion, one of the most delicate moments in a digital workflow is intraoral scan (IOS) procedures. The transfer of the three-dimensional position of the implant with respect to the bone or soft tissues is performed using scan bodies. Recent reviews have expressed a similarity in scan body results to traditional impressions [23,24]. Surely, in order to obtain a reproducible result, the clinician must always pay attention to the scan body position and the scanning technique. Furthermore, in the internal connections, the screwing torque of the scan bodies causes an abutment displacement that could affect the most extensive rehabilitations [25].

Computer-guided surgery planning also allows the preliminary computer-aided design of a screwed or cemented prosthesis according to the needs of the clinician and a predetermination of the aesthetic result. 

In all of the present study cases, cemented implant restorations were performed in order to adopt a one-time abutment protocol and therefore allow for an immediate seal of the prosthetic connection and a simpler implants axis management. 

A resorbable zinc-oxide-based cement was used to prevent peri-implantitis caused by eventual cement residue in the sulcus.

Abutments and CAD-CAM structures are known to offer better fitting than traditional cast components [26] due to a high level of reproducibility. Thanks to the possibility of predetermining the surgery according to the aesthetic needs and the greater precision of the milled elements, some clinicians have proposed to avoid the framework test [27].

In the present clinical study, a PMMA-test of the final restoration was carried out. Any chairside adjustments made on the PMMA were transferred to the technician. In fact, from the modified PMMA-test, it is possible to perform a STL. scan with the aim of milling the final prosthesis.

The present study has reported interesting clinical insights into the management of a fully digital workflow in cases of monoedentulism. Clearly, the whole process involves a margin of error that can be compensated for by segmenting the various workflow phases. 

In terms of prosthetic design, satisfactory aesthetic results were obtained. No occlusal plan modification was needed. These results were achieved thanks to the high precision of the impressions carried out by the clinician, the technician’s ability to predict the prosthesis’ exact height once activated on the implant connection and the ability to evaluate the periodontal ligament elasticity through the dental arch overlapping [28].

However, the positive outcomes of the present study have to be balanced with the reduced number of cases and the lack of randomization of patients in different protocols.

The present study demonstrates how it is predictably possible to complete a single implant rehabilitation in less than 4 months and 7 sessions. Not only does it represent an economic advantage for clinicians, but it also benefits patients by reducing rehabilitation operating sessions and waiting times. Digital dentistry also minimizes the risk of intra and post-operative complications [29]. Minimally invasive procedures allow us to treat patients with more complex systemic problems and to increase their quality of life [30,31].

In fact, a digital workflow and digital impressions, as documented by Bishiti et al. [32], are perceived as more comfortable and less invasive by patients.

Despite the positive outcomes of the present study, future investigations are needed to compare different fully digital protocols in monoedentulous. 

Furthermore, a technical and technological implementation of the protocols for multiple and total edentulousness rehabilitation is desirable. In fact, there is still no clear consensus in the literature regarding full-digital protocols reproducibility and precision in complex cases. However, a recent systematic review determined that a similar accuracy is reached when implants are inserted both in single- and partial edentulous following computer-aided surgery [33]. At the same time, a similar accuracy was obtained using an analogic or digital workflow for prosthetic restorations [34,35].

The limitations of this study are represented by the small sample of patients taken into consideration and the lack of a randomized case-control study design. This would allow us to compare all of the pros and cons of the full digital workflow both in terms of clinical advantages, costs and benefits for the dental facility.

Many of the cases taken into consideration concern implants in the aesthetic area. For this reason, future studies may consider a full-digital workflow integrating facial scanners as proposed by Raffone et al. [36,37] to increase the degree of communication and planning with the dental technician and the patient.

The integration of digital protocols within daily clinical practice represents the present and future of dental clinics. Digital technologies may seem to not be available to everyone due to the learning curve necessary to bring them into daily practice. However, the increasingly accessible costs of the technologies make it possible to set up a workflow that allows for the managing of more patients in relatively faster times. In addition, thanks to digital workflows, it is possible to increase the level of documentation of the cases, both to improve treatment plans and for medical–legal purposes.

Finally, future research will also be necessary to evaluate the digital workflow mentioned in terms of the quantity of data acquisition of .DICOM and .STL files, of their overlap to perform the best-matching and the degree of precision and fitting of the definitive prosthetic manufacts.

## 5. Conclusions

With digital protocols and digital planning, a reduced number of clinical sessions was registered. Continuous access to CAD-CAM projects allowed for a simplified process in managing eventual changes by copying and pasting phases, for example, from provisional to definitive prostheses. However, given the small number of cases registered for the present study, further studies should be considered and there should be further investigation on a full-digital workflow in complex cases where an implant replacement of multiple elements is required.

## Figures and Tables

**Table 1:** Demographic information.

Patients	Age (y)	Sex (M/F)	Site	Incisors	Canine	Premolar	Molar
19	Mean: 47.7 SD: 13.6	6/19	Mandible: 4 Maxilla: 15	12	2	5	3

## Data Availability

Not applicable.
